# Burning mouth syndrome: updates on pathogenesis and diagnostic algorithms

**DOI:** 10.22514/jofph.2025.064

**Published:** 2025-12-12

**Authors:** Federica Canfora, Elena Calabria, Niccolò Giuseppe Armogida, Giulia Ottaviani, Michele Davide Mignogna, Gianrico Spagnuolo, Daniela Adamo

**Affiliations:** ^1^Department of Neurosciences, Reproductive Sciences and Odontostomatology, University of Naples “Federico II”, 80131 Naples, Italy; ^2^Department of Health Sciences, School of Dentistry, University Magna Graecia of Catanzaro, 88100 Catanzaro, Italy; ^3^Department of Medicine, Surgery and Health Sciences, University of Trieste, 34129 Trieste, Italy; ^4^Therapeutic Dentistry Department, Institute for Dentistry, Sechenov University, 119991 Moscow, Russia; ^5^Department of Life Science, Health and Health Professions, Link Campus University, 00165 Rome, Italy

**Keywords:** Burning mouth syndrome, Dysesthetic sensation, Xerostomia, Anxiety, Depression, White matter change

## Abstract

Burning Mouth Syndrome (BMS) is a complex, chronic neuropathic orofacial pain disorder characterized by a persistent burning or dysesthetic sensation in the oral cavity without an identifiable organic cause. Accurate diagnosis and effective management of BMS pose significant challenges to clinicians, necessitating a comprehensive and multidisciplinary approach. This review delves into BMS’s pathogenesis and diagnostic algorithms, highlighting the latest advancements in understanding the underlying mechanisms and diagnostic strategies. Utilizing specific diagnostic algorithms assists clinicians in assessing and selecting appropriate treatment strategies, thereby minimizing diagnostic delays. These algorithms are crucial for excluding other causes of oral burning by focusing on symptomatology, patient history, and clinical examination. They involve ruling out oral infections, nutritional deficiencies, hormonal imbalances, autoimmune disorders, and medication side effects as potential causative factors. Supporting the diagnostic process, additional tests such as blood tests (including a thrombophilic panel and Neuron-Specific Enolase), neurosensory assessments, neuroradiological examinations, and evaluations of psychological profiles and cognitive function may be employed. Neurosensory assessments and neuroradiological examinations can provide insights into possible neuropathic causes, while psychological and cognitive assessments can identify any psychological factors and the extent to which cognitive decline may contribute to the condition. A comprehensive diagnostic approach not only aids in the accurate identification of BMS but also helps differentiate it from other oral conditions with similar presentations. This thorough evaluation is essential for developing a tailored treatment plan that addresses each patient’s specific needs, ultimately improving clinical outcomes and enhancing the quality of life for individuals suffering from BMS.

## 1. Introduction

Burning mouth syndrome (BMS), is a multifaceted idiopathic orofacial disorder characterized by persistent, chronic, and spontaneous pain. Patients often describe this discomfort as an oral burning sensation, lasting for over three months, without any identifiable local or systemic pathological changes. Although the tongue is frequently affected, any intraoral site may experience these distressing sensations [1]. Historically, this condition has been referred to by various terms, such as stomatodynia, glossodynia, burning tongue, oral dysesthesia, and complex oral sensitivity disorder—reflecting the broad spectrum of symptoms experienced by patients [2, 3, 4]. Moreover, individuals with BMS generally present with a range of additional intra-oral and extra-oral symptoms, further complicating and delaying the diagnostic process [5].

In recent years, a significant conceptual shift has emerged regarding the classification of BMS. While traditionally labeled as a “syndrome” due to its heterogeneous symptomatology—including burning sensations, xerostomia, dysgeusia, and other additional oral symptoms—this terminology has been increasingly questioned. The term “syndrome” implies a consistent cluster of clinical features that recur across most patients, yet in BMS, only the burning sensation appears to be reliably present, while other associated symptoms vary widely in intensity and presence. This inconsistency has prompted several experts in the field to advocate for a redefinition of BMS as a “disorder” rather than a “syndrome”. A recent Delphi consensus among international orofacial pain specialists supported the adoption of the term Burning Mouth Disorder (BMD) to more accurately reflect the clinical and pathophysiological complexity of the condition [6]. This terminology emphasizes the chronic neuropathic nature of the pain and the exclusion of other identifiable causes, aligning with the latest International Classification of Diseases (ICD-11) classification which categorizes BMS under “chronic primary pain” conditions [7].

Concurrently, an expanded diagnostic framework has been proposed to encompass patients presenting with dysaesthetic symptoms (such as tingling, taste disturbance, xerostomia, numbness, and itching) and perceptual symptoms (such as intraoral foreign body sensation, altered perception of tongue size or color, and sialorrhea), but without burning sensations, yet sharing key clinical features with BMS. The term Oral Dysaesthetic and Perceptual Disorder (ODPD) has been introduced to define this under-recognized subgroup, underscoring the need to broaden diagnostic criteria to better capture and manage such phenotypes [8].

In this review, we retain the term BMS for consistency. However, we fully acknowledge the evolving nosology that favors the more precise designation of BMD and the recognition of emerging categories such as ODPD. These developments have important implications for improving diagnostic accuracy, guiding future research, and optimizing clinical management.

Despite ongoing research, the pathogenesis of BMS remains elusive. Current evidence suggests the involvement of central and peripheral neuropathy [9, 10], along with potential contributions from endocrinological and psychosocial factors [11, 12, 13], in the development and perpetuation of this enigmatic condition. Recognizing the intricate interplay of these factors is essential in developing effective strategies for diagnosis, treatment, and management.

This review aims to comprehensively present the multifaceted nature of BMS, exploring its clinical manifestations, potential underlying mechanisms, and the challenges encountered in both diagnosis and treatment. A pivotal focus of this review is on proposing and discussing specific diagnostic approaches to assist clinicians in differential diagnosis, and in the formulation personalized treatment plans for BMS patients. By synthesizing existing knowledge and presenting a structured approach to understanding and addressing BMS, this review endeavors to deepen the understanding of this complex affection and enhance clinical practices.

## 2. Epidemiology: prevalence, age, and sex distribution

The worldwide prevalence of BMS is estimated at 1.7% in the general population, rising to 7.7% among dental patients in clinical settings. However, precise prevalence data can vary significantly due to differences in populations, geographic regions, and diagnostic criteria [14]. Additionally, a population-based study by Kohorst *et al*. [15] reported a prevalence of 0.11% for BMS, with a higher incidence observed in women over the age of 60. In the general population, BMS prevalence is higher in Europe (5.5%) and North America (1.1%), and lower in Asia (1%). Conversely, in clinical settings, the prevalence is higher in Asia (8.9%) compared to South America (6.1%) and Europe (6.5%). These variations highlight the impact of geographic location on BMS prevalence [14].

Gender-specific analysis revealed a prevalence of 1.1% in females, significantly higher than the 0.38% observed in males in the general population. Consequently, the female-to-male ratio is approximately 3:1 in population-based studies, whereas in clinical settings it may reach 6:1 to 9:1, highlighting a notable gender disparity in BMS prevalence. This disparity may be related to physiological and behavioral differences [14].

Interestingly, recent large cohort studies suggest a shift in the onset of BMS towards older ages, with the disease manifesting predominantly around 65 years, emphasizing the evolving landscape of BMS epidemiology in the context of an aging population [14]. An age-specific analysis indicated a higher incidence of BMS among individuals aged 50 and above (3.31%) compared to those under 50 (1.92%). This trend may be attributed to various factors that differ across the countries, including hormonal changes, nutritional deficiencies, differences in socioeconomic and medical conditions or the cumulative effects of environmental factors over time (Table 1, Ref. [14]). This also highlights important methodological differences across studies.

**Table 1.** Prevalence of burning mouth syndrome (BMS) according to Wu *et al*. [14].

		Estimated prevalence
Prevalence category		
	General prevalence	1.73
	Clinical prevalence	7.72
Country-specific general prevalence		
	Europe	5.5
	North-America	1.1
	Asia	1.05
Country-specific clinical prevalence		
	Asia	8.96
	Europe	6.46
	South America	6.05
Gender-specific general prevalence		
	Female	1.15
	Male	0.38
Age-specific prevalence (yr)		
	<50	3.31
	>50	1.92

## 3. Aetiopathogenesis

### 3.1 Neuropathological insight

The pathogenesis of BMS remains an enigma and is not fully understood. Nevertheless, there is a widespread consensus that the etiology of BMS is intricate and multifactorial, with different patients exhibiting various combinations of triggers and perpetuating factors [16, 17]. Neurological, psychological, and hormonal factors are extensively recognized as potential contributors to the development of this condition [12, 18, 19]. Additionally, local factors, systemic conditions, and certain medications have been implicated in provoking secondary burning sensations in the oral cavity [20, 21]. A comprehensive understanding of these diverse factors, their distinct characteristics, and interactions is imperative for accurate diagnosis and, subsequently, for developing more effective treatment strategies.

The trigeminal nerve transmits nociceptive sensory information to various regions of the central nervous system, which process both the sensory-discriminative and affective-emotional aspects of orofacial pain [22] (Fig. 1). Recent neuroimaging advancements have significantly enhanced our comprehension of the pathophysiological mechanisms underlying BMS [23, 24]. These studies have provided consistent evidence that BMS is a neuropathic disorder involving both the peripheral (PNS) and central nervous systems (CNS), highlighting the role of the trigeminal nerve in transmitting abnormal pain signals in BMS patients [25, 26]. In this scenario, both peripheral and central sensitization, characterized by an increased responsiveness of peripheral neurons and an enhanced excitability of central neurons may further contribute to persistent pain [27] (Fig. 2).

**Fig. 1.** Anatomy of the trigeminal nerve, sensory nuclear complex, and ascending pain pathways. The fifth pair of cranial nerves (V), known as the trigeminal nerve, is divided into three main branches: ophthalmic (V1), maxillary (V2), and mandibular (V3). Nociceptive sensory information from the areas innervated by the trigeminal nerve is transmitted via trigeminal afferents to second-order neurons in the trigeminal sensory nuclear complex (TSNC) located in the brainstem and the upper cervical spinal cord (C1–C2). The TSNC comprises the principal sensory nucleus (PSN) and the spinal trigeminal nucleus (SpTN), which is functionally divided into three subnuclei: oralis (Or), interpolaris (Ip), and caudalis (Ca). The trigeminal nuclei are considered equivalent to the dorsal horn of the spinal cord for orofacial pain. The Ca and C1–C2 regions are primary sites for synaptic integration of sensory inputs from craniofacial tissues. Three main areas of the central nervous system (CNS) receive nociceptive input from the SpTN and C1–C2 regions: the ventral posteromedial thalamic nucleus (VPM), the medial thalamic nuclei (MTN), and the parabrachial nucleus (PBN). Nociceptive neurons in the VPM, which receive input from the orofacial region, send axons to neurons in the primary (S1) and secondary (S2) somatosensory cortices. These neurons are involved in the sensory-discriminative perception of pain (intensity and localization). In contrast, those in the MTN and PBN project first to limbic cortices, such as the anterior cingulate cortex (ACC) and insula (I), and subsequently to the S2, and are involved in the affective and emotional aspects of pain. OrN: Nucleus Oralis; IpN: Nucleus Interpolaris; CaN: Nucleus Caudalis; VPMTN: Ventral Posteromedial Thalamic Nucleus; IC: Insular Cortex; CSS: Somatosensory Cortex; MSC1/C2: Mesencephalic Nucleus 1 and 2.

**Fig. 2.** Peripheral and central sensitization of pain. Damage to the trigeminal nerve or inflammation can induce hyperexcitability of primary afferent neurons of the trigeminal system, which release excitatory neurotransmitters (glutamate (Glu), substance P (SP), calcitonin gene-related peptide (CGRP), brain-derived neurotrophic factor (BDNF), and ATP) that activate normally silent glutamate receptors (NMDA receptors) present on second-order neurons. In this context, C fibers and A-delta fibers play crucial roles. C Fibers: These unmyelinated fibers are responsible for transmitting slow, dull, and aching pain. They release excitatory neurotransmitters such as substance P and CGRP, which contribute to the development of hyperalgesia by promoting inflammation and sensitizing neurons. A-Delta Fibers: These thinly myelinated fibers are involved in transmitting fast, sharp pain. They release neurotransmitters like glutamate, which can activate NMDA receptors on second-order neurons and contribute to central sensitization. Additionally, tissue damage or inflammation activates the release of ATP, chemokines, and fractalkine from primary afferent neurons, which in turn activate satellite glial cells (microglia and astrocytes). Activated microglia (M1) release a range of cytokines such as TNFα, IL-1β, IL-6, NGF, and BDNF, while activated astrocytes release CCL2, glutamine, and NF-κB, further contributing to neuronal hyperexcitability by increasing the expression of pain receptors TRPV1, TRPA1, and P2X3. Under normal conditions, inhibitory interneurons continuously release GABA to decrease the excitability of nociceptive neurons and modulate nociceptive transmission (inhibitory tone). Following damage or inflammation, this inhibition can be lost (disinhibition), resulting in hyperalgesia. ATP: Adenosine Triphosphate; TNFα: Tumor Necrosis Factor-alpha; NGF: Nerve Growth Factor; IL-1β: Interleukin-1β; IL-6: Interleukin-6; CCL2: Chemokine CCL2; NF-κB: Nuclear Factor Kappa; TRPV1: Transient Receptor Potential Vanilloid 1; TRPA1: Transient Receptor Potential Cation Channel A1; P2X3: Purinergic Receptor P2X3; NK1R: Neurokinin 1 Receptor; GABA: Gamma-Aminobutyric Acid; Ca++: Calcium Ions; Cl−: Chloride Ions; NC: Caudal Nucleus; MS-C1C2: Upper Cervical Spinal Cord.

Understanding the involvement of the trigeminal nerve and its pathways in the CNS is crucial for developing targeted treatments for the sensory and emotional dimensions of BMS. BMS can be defined as a nociplastic pain because it is characterized by altered nociception in the absence of clear evidence of actual or threatened tissue damage that would activate peripheral nociceptors or indicate a disease or lesion of the somatosensory system causing the pain. This classification underlines the absence of visible signs or detectable disorders that typically accompany other types of pain, thereby situating BMS within the realm of conditions where the pain arises primarily from dysfunctions in the pain processing systems. Studies have shown that BMS is associated with dysfunction of the pain modulatory system, particularly the descending pain inhibition, where altered brain function can be demonstrated through neurophysiological tests and imaging, revealing an atypical response to painful stimuli without the usual physical causes [28, 29].

### 3.2 Central neuropathy

Central neuropathy in BMS involves several key alterations in the CNS, including structural and functional changes that indicate brain hypoactivity, impacting pain modulation and sensory processing [23, 24, 26, 30].

#### 3.2.1 Striatal dopamine, substantia Nigra and midbrain raphe alterations

Striatal dopamine dysfunction, particularly depletion in the putamen, impairs pain inhibition in the trigeminal brainstem complex. Transcranial sonography reveals unique abnormalities, such as hypoechogenicity in the substantia nigra and midbrain raphe, suggesting nigrostriatal dopaminergic hypofunction. Indeed, a hypofunction in the nigrostriatal dopaminergic pathway, particularly in the basal ganglia and sensory cortex, has been suggested as a cause of reduction of endogenous pain inhibitory control. Specifically, the increased availability of dopamine D2 receptors, reflecting dopamine depletion, may contribute to chronic neuropathic pain within the trigeminal distribution [31, 32].

#### 3.2.2 Brain activation and connectivity

Advanced neuroimaging techniques, such as functional Magnetic Resonance Imaging (fMRI), have revealed a spectrum of changes affecting both structural and connectivity aspects of the brain’s pain matrix in BMS patients. These patients exhibit decreased activation, especially in the thalamus, alongside an expansion in hippocampal grey matter volume (GMV) and a reduction in the medial prefrontal cortex [33]. Further supporting this, studies have established a correlation between pain intensity and reductions in grey matter concentration (GMC) and GMV in BMS patients compared to control groups. Specifically, these reductions are observed in the thalamus, cingulate gyrus, cerebellar lobules, insula/frontal operculum, inferior temporal area, primary motor cortex, and medial and dorsolateral prefrontal cortex [24, 34]. Similar reductions in GMV in the prefrontal cortex have been documented in various chronic pain conditions, such as back pain, trigeminal neuralgia, temporomandibular disorder, and functional dyspepsia, as well as in depression (D) and anxiety (A) [35].

Moreover, BMS patients show distinct brain activation patterns, including increased connectivity in the left insula, right amygdala, and right lateral orbitofrontal cortex, and decreased connectivity between the bilateral medial prefrontal cortex and the amygdala. These changes, which correlate with BMS duration, along with reduced thalamic activity, underscore the thalamus’s critical role in pain signal transmission and BMS pathogenesis [30]. Furthermore, reductions in cerebral blood flow in the middle temporal gyrus and insula have been documented, highlighting the role of altered neurovascular dynamics in BMS [35].

#### 3.2.3 White matter hyperintensities

Recent research has identified an elevated incidence of White Matter Hyperintensities (WMHs) in specific brain regions of BMS patients, particularly in the frontal, parieto-occipital, and temporal areas. These WMHs, visible on MRI through T2-weighted or Fluid Attenuated Inversion Recovery (FLAIR) sequences, are early indicators of brain vulnerability and may signify accelerated brain aging (Fig. 3).

**Fig. 3.** White matter hyperintensities in a burning mouth syndrome (BMS) patient. Axial T2-weighted fluid-attenuated inversion recovery (FLAIR) images from a 63-year-old male BMS patient. (A) Axial image at supraventricular level shows multiple hyperintense gliotic foci predominantly in the frontal and parietal white matter. (B) Axial image at the level of the temporal lobes highlights hyperintense foci (white arrows) in the temporal white matter, particularly involving the mesial temporal structures.

They are associated with cerebral small vessel arteriolosclerosis and potential links to vascular dementia and neurodegenerative diseases like Alzheimer’s. The connection between chronic orofacial pain, including BMS, and WMHs remains debated, with uncertainty about whether WMHs contribute to the onset of chronic pain or exacerbate BMS symptoms [36]. A recent study by Kato *et al*. [23] used Diffusion Tensor Imaging (DTI) and Neurite Orientation Dispersion and Density Imaging (NODDI) to evaluate white and gray matter abnormality in BMS. The study included 14 BMS patients and 11 healthy controls, analyzing various metrics such as fractional anisotropy (FA), mean diffusivity (MD), axial diffusivity (AD), radial diffusivity (RD), intracellular volume fraction (ICVF), and isotropic volume fraction (ISO). Results showed that BMS patients exhibited higher FA and ICVF and lower MD and RD in widespread white matter areas, as well as higher ISO and lower MD and RD predominantly in the amygdala. These findings suggest microstructural changes in both white and grey matter, indicating alterations in myelination, astrocytic hypertrophy, and neuroinflammation, reflecting the complex pathology of BMS [23].

### 3.3 Peripheral neuropathy

#### 3.3.1 Peripheral nerve fiber alterations

BMS is linked to dysfunction of peripheral trigeminal fibers and their connectivity with the brainstem in about 20–30% of the patients [37]. Multiple studies have identified alterations in the nerve fibers of the tongue’s mucosal lining in BMS patients [25, 38]. These studies have reported a decreased density of small intraepithelial and subpapillary nerve fibers, while larger subepithelial fibers remain intact. This significant reduction in intraepithelial nerve fiber density suggests the involvement of trigeminal small-fiber sensory neuropathy or atrophy of the oral mucosa. However, current histological evidence does not conclusively attribute these reductions specifically to Aδ or C fibers. Some studies hypothesize that degeneration of Aδ fibers may lead to disinhibition of C fibers, contributing to the persistent burning pain characteristic of BMS [37, 39]. Additionally, sensory alterations in BMS correlate with functional deficits in other cranial nerves, such as facial and olfactory nerves. This dysfunction is supported by quantitative sensory testing (QST) and neurophysiological recordings, indicating abnormal responses to thermal and pain-evoked potentials, supporting the role of peripheral neuropathy in BMS. Trigeminal tactile Aβ fiber hypofunction has also been identified through electrical thresholds analysis following blink reflex stimulation in BMS patients [37, 38]. Taste perception alterations in BMS patients have been a subject of investigation, with studies yielding varying results. Some research suggests that BMS patients experience disturbances in taste perception, potentially linked to dysfunction of the chorda tympani nerve, which plays a crucial role in taste sensation. These disturbances may manifest as reduced sensitivity to sweet, sour, salty, and bitter tastes, implicating the involvement of small afferent nerve fibers in the pathophysiology of BMS [40]. However, recent well-controlled studies have challenged this association. For instance, Kolkka *et al*. [41] conducted a study comparing BMS patients to age- and gender-matched control subjects and found no significant differences in taste perception or salivary composition between the groups. This suggests that taste alterations may not be a consistent feature of BMS and highlights the need for further research to clarify these findings.

#### 3.3.2 Receptor dysregulation

Receptor dysregulation has been implicated in the pathophysiology of BMS. Immunohistochemical studies have demonstrated increased expression of transient receptor potential vanilloid 1 (TRPV1) and cannabinoid receptor 1 (CB1), along with decreased expression of cannabinoid receptor 2 (CB2), in the oral mucosa of BMS patients [42]. These receptors are involved in nociceptive signaling and may contribute to peripheral sensitization. Voltage-gated sodium channels (VGSCs), particularly Nav1.7 and Nav1.9, have also been investigated, although studies have not found statistically significant differences between BMS patients and healthy controls [43]. Purinergic receptors (P2X3) are known to be expressed in gustatory fibers of the chorda tympani rather than in trigeminal sensory fibers, suggesting it may be more relevant to taste signaling than to nociception in BMS [44]. Additionally, non-neuronal cells such as fibroblasts, Schwann cells, and astroglia can contribute to pain sensitization by releasing nerve growth factor (NGF), which increases during inflammation and enhances nociceptor responsiveness [45].

#### 3.3.3 Recent advance in peripheral neuropathy

Recently, a new hypothesis for the pathogenesis of BMS has been proposed. This hypothesis revolves around the uncontrolled activation of specific calcium-permeable transmembrane channels, found within the intraoral mucosal nerve fibers, potentially triggered by an increase in reactive oxygen species (ROS) or impairments in anti-apoptotic pathways, leading to oxidative stress-mediated apoptosis signaling. This cascade of events likely results in the depolarization of nerve endings, generating action potentials that may be interpreted centrally as pain [46]. Neuron-specific enolase (NSE), a glycolytic enzyme predominantly found in neurons and neuroendocrine cells, has been investigated as a potential biomarker of neuronal damage or dysfunction in various neuropathic conditions. In the context of BMS, elevated levels of NSE have been reported in some studies, suggesting an underlying neuropathic component in a subset of patients [47]. Increased NSE expression may reflect peripheral or central sensitization processes or subtle neuronal injury within the trigeminal system [48]. Although current evidence remains limited and preliminary, NSE could contribute to the identification of neurobiological alterations in BMS and may, in the future, aid in the stratification of patients and the development of targeted therapeutic approaches. Further studies are warranted to validate its clinical utility in this setting.

## 4. Stress and neuromodulation

Stress plays a significant role in the pathogenesis and symptom modulation of BMS. Beyond immediate adaptive responses via catecholamines and cortisol, chronic stress induces long-term neuroendocrine, immune, and epigenetic changes that affect pain perception. These include the release of pro-inflammatory cytokines, altered neural plasticity, and modulation of gene expression through mechanisms like DNA methylation and microRNAs, potentially facilitating central sensitization and hyperalgesia. Experimental studies have shown that prolonged stress can suppress dopaminergic activity in the nucleus accumbens (NAc) and alter tyrosine hydroxylase expression in the ventral tegmental area (VTA), impairing dopamine synthesis and signaling in regions involved in orofacial pain, such as the NAc, dorsal striatum, and amygdala [49]. This highlights the bidirectional relationship between stress and pain processing in BMS. Moreover, BMS patients appear particularly vulnerable to stress-induced hyperalgesia. During the COVID-19 pandemic, they exhibited increased symptom severity and distress compared to controls, including higher levels of intrusive cognitions and emotional dysregulation. Chronic stress also affects neuroendocrine biomarkers: elevated salivary cortisol and α-amylase levels have been observed in BMS patients, with reductions correlating with symptom improvement. These markers may serve as objective indicators of stress-related exacerbation in BMS.

## 5. Interplay of mood disorders and burning mouth syndrome

It is widely acknowledged that a dynamic interplay of psychological and neurological mechanisms plays a crucial role in the syndrome’s onset and progression, as suggested by the biopsychosocial model (Fig. 4).

**Fig. 4.** The biopsychosocial model of pain. This figure illustrates the integration of biological, psychological, and social factors in understanding the experience of pain. The model emphasizes that pain is influenced by a complex interplay between physiological processes (biological factors), mental and emotional states (psychological factors), and social contexts including relationships, socioeconomic status, and cultural influences (social factors). The interconnected circles represent how each dimension impacts and interacts with the others, highlighting the need for a comprehensive approach to patient care and intervention.

Mood disorders (MDs), such as A and D, and certain aberrant personality traits are often linked with conditions like fibromyalgia, irritable bowel syndrome, chronic fatigue, and notably, BMS. A pivotal study by Taiminen *et al*. [50] revealed that over half of the patients with BMS had experienced at least one Axis I psychiatric disorder during their lifetime, with major D being the most prevalent. Notably, a significant proportion of these psychiatric conditions preceded the onset of BMS symptoms, suggesting a potential contributory role in the pathogenesis of the syndrome. Additionally, a considerable number of patients exhibited Axis II personality disorders, particularly those within cluster C, characterized by anxious and fearful behaviors. These findings underscore the importance of comprehensive psychiatric evaluation in patients presenting with BMS symptoms [50].

### 5.1 The chicken or the egg: causality dilemmas

A significant challenge in understanding BMS lies in determining whether MDs are a cause or a consequence. Until now, it is controversial whether A and D act as driving forces in the development of BMS or, conversely, if the onset of BMS triggers a decline in mental health, potentially leading to these mood disorders over time [51]. Recent evidence indicates that in approximately 80% of cases, MDs often precedes the onset of BMS, suggesting that psychological impairments appear to be potential contributing factors to BMS’s development. Specifically, patients with A and D are at a higher risk of developing BMS compared to those without such conditions. Interestingly, this correlation does not extend to bipolar disorder, which does not show a significant association with BMS [13]. A noteworthy finding is that A seems to predispose individuals to BMS at an earlier stage than D, suggesting a heightened vulnerability in anxious individuals. This leads to a hypothesis that A might induce secondary D, which in turn could contribute to the manifestation of pain, reinforcing the idea that BMS might be a somatic expression of depressive disorders [13].

Interestingly, a comprehensive nationwide cohort study by Kim *et al*. [18] found that patients with BMS were at an increased risk of developing D and A compared to individuals without BMS. This association persisted even after adjusting for sociodemographic characteristics and comorbid conditions, suggesting a potential predisposition to D and A in BMS patients [18]. Although the exact nature of this relationship remains unresolved, analysis of the studies indicates that the relationship between mood and pain is inherently bidirectional, with each factor influencing the other. Chronic pain and discomfort associated with BMS may exacerbate psychological distress, creating a challenging cycle that complicates both diagnosis and treatment. Conversely, the persistence of mood disorders can amplify the perception of pain, further entrenching this complex interplay between mood and pain [52].

### 5.2 Sleep disturbances and BMS: a bidirectional relationship?

MDs are closely linked with sleep disturbances (SD), which can be both symptoms and contributing factors. Common sleep issues like insomnia, difficulties in falling or staying asleep, and hypersomnia (excessive sleepiness) are pivotal in diagnosing MD. Poor sleep can increase irritability and exacerbate feelings of sadness or hopelessness, potentially worsening mood disorders. In some cases, SD may precede and trigger mood disorders in susceptible individuals. Long-term sleep disruption can alter brain chemistry and function, predisposing individuals to mood dysregulation. The co-occurrence of SD, with or without MD, may further exacerbate conditions like BMS.

In a case-control multicenter study, 78.8% of BMS patients experienced poor sleep, showing also an overall poorer sleep quality, higher levels of daytime sleepiness, and increased D and A compared to healthy controls. This highlights a significant correlation between BMS, poor sleep quality, A, and D. Possibly, poor sleep, by exacerbating negative moods, may create a feedback loop with the pain experienced by BMS patients [53, 54].

### 5.3 Neurobiological insights among BMS, mood disorders and sleep disturbances

MDs, SD, chronic orofacial pain and BMS share overlapping neurophysiological mechanisms. These include dysregulation of the hypothalamic-pituitary-adrenal (HPA) axis, altered monoaminergic neurotransmission (such as serotonin and dopamine), and dysfunctions in brain regions like the anterior cingulate cortex, insula, and prefrontal cortex [30, 55, 56]. Functional MRI studies further support this overlap, revealing structural and connectivity changes common to BMS, D and SD [24, 30].

#### 5.3.1 Brain changes and connectivity

Key brain areas, including the prefrontal cortex, amygdala, and hippocampus, exhibit notable changes in size and activity in individuals with mood disorders and chronic orofacial pain. These changes impact how we regulate emotions and perceive pain. For instance, increased activity in the default mode network (a brain network active during rest) is observed in BMS and MD. This could reflect a heightened focus on internal thoughts and pain sensations. Furthermore, there is a notable dysregulation in how emotions are processed, suggested by increased connectivity between the amygdala and other limbic regions. The insula, another critical area for emotional and internal bodily sensation processing, also shows increased connectivity. This change is believed to alter emotional and bodily awareness. On the other hand, a decrease in connectivity between the prefrontal cortex and regions processing pain signals has been observed. This suggests a disruption in both emotion regulation and cognitive processing in both conditions. Other brain areas, like the thalamus, somatosensory cortex, and hippocampus, also show altered connectivity. These changes in brain connectivity and function could lead to an intensified perception of pain and its prolonged presence, potentially affecting memory processes and the regulation of stress responses [30].

#### 5.3.2 Neurotransmitters imbalance and genetic factors

Recent studies have highlighted a compelling link between neurotransmitter imbalances and the co-occurrence of MD and BMS, finding that BMS patients exhibit dysregulated levels of key neurotransmitters, including serotonin, dopamine, and noradrenaline, which are crucial for pain perception and modulation. This neurochemical basis for BMS is supported by the varied success of antidepressants in alleviating BMS symptoms [57, 58]. Research into the role of dopamine, its receptors, and genetic polymorphisms has shown significant overlap in the dopaminergic system’s involvement in both pain and mood regulation. Dopamine, essential for the brain’s reward system, influences pleasure, mood regulation, and pain perception through direct and indirect pathways. Alterations in dopamine receptor functionality and levels might underlie the sensory issues characteristic of BMS [16]. Genetic studies have further explored these connections, especially the associations between dopamine receptor gene polymorphisms 2 and 4 (DRD2 and DRD4) and major depressive disorder. These polymorphisms can affect dopamine receptor expression and functionality, impacting mood regulation [59]. In BMS patients, dysregulation of these receptors, especially in pain-related brain areas, has been documented. Kolkka *et al*. [26] investigated the 957C>T polymorphism of the DRD2 gene in BMS patients, finding that the 957TT genotype is linked to higher pain thresholds, greater pain interference in daily activities, and more intense suffering. These findings collectively suggest a potential genetic predisposition in certain BMS and mood disorder patients related to dopamine pathway functionality.

#### 5.3.3 Neuroinflammation and HPA axis dysregulation

Research is increasingly focusing on neuroinflammation as a common pathway linking chronic pain conditions with psychiatric comorbidities [60]. The role of pro-inflammatory cytokines in altering neurotransmitter systems and neuronal activity is well-documented, influencing both emotional states and pain sensitivity. Specifically, cytokines, such as interleukin 6 (IL-6) and tumor necrosis factor α (TNF-α), can modulate brain function and induce depressive symptoms, particularly through their effects on neurotransmitter metabolism and brain signaling pathways. Indeed, these cytokines may disrupt the synthesis and reuptake of neurotransmitters, impacting both pain perception and mood [61]. Although elevated salivary levels of IL-6 and TNF-α have been reported in BMS patients by Al-Maweri *et al*. [62], findings across studies remain inconsistent. A recent prospective case-control study by Moreau *et al*. [63] found no significant differences in salivary cytokines, steroid hormones, or neuroinflammatory markers between BMS patients and healthy controls, suggesting that systemic inflammation may not play a central role in BMS pathogenesis. This connection is attributed to the HPA axis’s role in mediating stress responses and its influence on neuroendocrine and immune functions. It suggests that HPA axis dysregulation might contribute to BMS pathogenesis, potentially through stress-induced neurogenic inflammation or altered pain perception [64]. Indeed, prolonged activation of the HPA axis can lead to cortisol overproduction, affecting neurotransmitter systems, notably serotonin and dopamine, crucial in both pain and mood regulation. Additionally, increased levels of pro-inflammatory cytokines, associated with both BMS and mood disorders, can further dysregulate the HPA axis, creating a feedback loop that may exacerbate these conditions [65].

#### 5.3.4 Central sensitization

The alterations in CNS processing, commonly observed in MD and BMS, may lead to a state of central sensitization. This condition is typified by the heightened responsiveness of nociceptive neurons in the CNS to both normal and subthreshold afferent input, potentially resulting in amplified pain perception in BMS. Furthermore, central sensitization is also linked to MD, given its significant impact on neural networks that regulate emotions [26, 66] (Fig. 2). It is important to note that the neurobiological mechanisms underpinning central sensitization involve both changes in synaptic efficacy and an increase in neuronal excitability within the CNS. These alterations can influence key neurotransmitter systems, such as serotonin and norepinephrine, which play vital roles in regulating both pain and mood [67].

## 6. Hormonal imbalance in BMS

Hormonal dysregulation, especially involving sex steroids, has been considered a possible contributing factor in BMS largely based on its higher prevalence in peri- and postmenopausal women. While early studies suggested that reduced estrogen may impact peripheral nerve sensitivity and salivary function, recent data remain inconclusive. For example, a recent prospective case-control study found no significant differences in salivary estrogen levels between BMS patients and healthy controls [63], while other investigations have reported reduced salivary estradiol in symptomatic postmenopausal women [68]. Experimental research has also proposed that menopause-related estrogen decline could increase pain sensitivity through upregulation of TRPV1 and NGF pathways, potentially sensitizing peripheral nerves [69]. However, such mechanisms remain largely theoretical and need further validation in clinical settings. Regarding adrenal steroids, data on dehydroepiandrosterone (DHEA) are inconsistent. Some studies suggest lower salivary DHEA levels in BMS patients, possibly reflecting chronic stress or adrenal fatigue, but other investigations have failed to confirm this association [70]. Overall, while hormonal fluctuations—particularly related to menopause—may contribute to BMS susceptibility, current evidence is mixed, and hormone replacement therapy has shown inconsistent results, suggesting that hormonal factors are likely part of a broader multifactorial etiology.

## 7. Association of systemic comorbidities and BMS

BMS patients frequently encounter a higher incidence of simultaneous medical comorbidities and consume more medications than controls. This scenario often results in an overall poorer health status, which can, in turn, intensify the symptoms and potentially accelerate the progression of BMS itself. MD, hypertension, hypercholesterolemia, hyperhomocysteinemia, hypothyroidism, and gastroesophageal reflux disease (GERD) are the most frequent comorbidities associated with BMS. Concerning co-occurring pain conditions, a systematic review by Moisset *et al*. [71] demonstrated that such comorbidities are relatively uncommon among individuals with BMS, thereby supporting the hypothesis that BMS may involve distinct pathophysiological mechanisms compared to other chronic pain disorders. Understanding the multifaceted nature of these comorbidities and their interactions with BMS is essential for developing a comprehensive and tailored treatment plan. This involves not only addressing the primary symptoms of BMS but also managing any underlying or concurrent conditions that may be contributing to the patient’s overall health challenges.

### 7.1 BMS and mood disorders

Several studies reported that MDs are the most frequently associated comorbidities in BMS [72]. Specifically, A, D, MDs, and medically unexplained extraoral physical symptoms (MUEPS) are the most commonly observed [53, 72, 73]. On one side, patients suffering from A and D are at an increased risk of developing BMS, with this risk being more pronounced in females and increasing with age. Notably, a prevalence of 80% of MDs has been observed among BMS patients, often preceding the development of BMS, suggesting that psychological factors might contribute to the pathogenesis of the disease [13]. On the other side, BMS patients may also be predisposed to A (odd ratio 2.64) and D (odd ratio 3.18) [72]. Furthermore, evidence suggests that individuals with BMS may be at increased risk for A and D, independent of sociodemographic characteristics and other comorbid conditions [17]. Considering this evidence, which supports the hypothesis of a bidirectional relationship between MDs and BMS, it is crucial to acknowledge that the co-occurrence of MDs and BMS tends to exacerbate both psychological symptoms and oral pain. BMS patients additionally show a high prevalence of SD, ranging between 78.8% and 90.2%. Interestingly, in almost half of the patients suffering from insomnia, the onset of this sleep disorder was observed on average 4 years before the BMS development [13, 53]. Moreover, in recent research on 500 BMS patients, MUEPS have been observed in 169 (33.8%) patients with a mean of 2.8 ± 1.50 symptoms for each patient. In this study, irritable bowel syndrome (48; 9.6%), fibromyalgia (36; 7.2%), tinnitus (32; 6.4%), and vulvodynia (21; 4.2%) were the most common MUEPS reported [73]. Interestingly, Leuci *et al*. [74] reported that vulvodynia is often linked with lower sexual desire, though no direct correlation with A, D, or sleep disorders was found. This finding may suggest that BMS might independently affect sexual desire, potentially due to dysfunction in the brain’s reward system [74]. Additionally, BMS patients may experience other psychological comorbidities like social phobia, cancerphobia, hypochondria, and neuroticism. However, bipolar disorder does not show a significant link to BMS [13].

### 7.2 BMS and vascular comorbidities: hypertension, hypercholesterolemia and hyperhomocysteinemia

Several vascular risk factors have been investigated about BMS, including hypertension, hypercholesterolemia, and hyperhomocysteinemia. Although evidence remains limited and somewhat heterogeneous, these conditions have been reported with increased prevalence in BMS populations, suggesting potential shared mechanisms or comorbid vulnerability.

Several studies have explored the association between hypertension and BMS, although findings remain inconsistent. A case-control study by Jin *et al*. [75] found no significant difference in hypertension prevalence between BMS patients and controls. In contrast, other studies have reported a higher prevalence of hypertension among BMS patients, particularly in older women. For instance, one study indicated that 51.2% of postmenopausal BMS women had hypertension compared to 30.4% of age-matched controls [76]. This suggests that certain subgroups, such as older or socioeconomically disadvantaged women, may be more vulnerable to comorbid cardiovascular conditions alongside BMS. Factors such as lower education level and unemployment were also associated with increased hypertension rates in BMS patients, pointing to a potential biopsychosocial component in disease expression.

As for hypercholesterolemia, although specific studies investigating its pathophysiological role in BMS are lacking, data from Adamo *et al*. [5] reported a prevalence of 38.6% among BMS patients, suggesting that lipid imbalance may frequently coexist with BMS. These findings underline the importance of evaluating cardiovascular risk factors during clinical assessment and management of BMS patients.

Elevated homocysteine levels have been observed in some patients with BMS, although findings are also still limited. Hyperhomocysteinemia has been associated with a wide range of systemic effects, including vascular and neurological complications, and has been proposed as a potential risk factor for peripheral neuropathy. In BMS, a study by Adamo *et al*. [77] reported a high prevalence of hyperhomocysteinemia (up to 73%), which may be related to the presence of white matter hyperintensities (WMHs) and cerebral small vessel disease. While the exact mechanisms remain unclear, these findings suggest a possible role of vascular dysfunction in the pathophysiology of BMS. However, further research is needed to establish whether elevated homocysteine levels are causally linked to BMS or reflect broader comorbid conditions.

### 7.3 BMS and thyroid disorders

The association between thyroid disorders and BMS is a subject of ongoing debate. While some researchers believe that thyroid alterations have minimal impact on BMS, others argue that they may contribute to symptoms. Notably, thyroid disorders are not currently a criterion for excluding BMS diagnosis. The thyroid gland produces hormones such as triiodothyronine (T3) and thyroxine (T4), which are crucial for tissue development, metabolism, and various functions and processes of the nervous system. Additionally, these hormones are involved in the maturation and specialization of taste buds. Consequently, a deficiency in thyroid hormones may contribute to dysgeusia (distorted taste perception) [78]. Given the high prevalence of taste disturbances in BMS, several researchers have explored this connection. Specifically, the prevalence of hypothyroidism largely varies, with a study showing a prevalence of hypothyroidism of 5.2% [79] and another of 13.6% [5]. On the other side, even a lower prevalence of hyperthyroidism has been reported, between 1% and 3.2% [5, 79]. Given that the majority of BMS patients are women and thyroid disorders predominantly affect women, it is challenging to definitively support the association between BMS and thyroid disorders based on current literature. However, a clinical study by Femiano *et al*. [80] reported improvement in BMS symptoms following correction of thyroid hormone dysfunction, supporting a possible therapeutic and diagnostic link between thyroid alterations and BMS [80].

### 7.4 BMS and gastroesophageal reflux disease (GERD)

The correlation between BMS and GERD is an area of growing interest in medical research. Until now, the classification systems for BMS have not ruled out gastrointestinal disorders, including GERD from the diagnostic criteria for BMS. Numerous studies suggest a potential link between GERD and BMS. However, a recent scoping review by Li *et al*. [81] indicates that the direct correlation and causal relationship between these two conditions still require clear demonstration. GERD is characterized by the backflow of stomach acid into the esophagus, often causing heartburn and can lead to oral burning sensations. This is presumably due to the irritation of nerve endings in the mouth by the refluxed acid, which may exacerbate BMS symptoms. Supporting this theory, observed lower oral pH values in GERD patients, suggest that gastric acid’s impact on the oral mucosa could be a contributing factor [82]. Additionally, many BMS patients have reported symptom improvement following treatment with proton pump inhibitors (PPIs), as noted in several studies [83, 84, 85]. This improvement further supports the hypothesis of a GERD-BMS connection. Furthermore, a high prevalence of oral pepsin saliva was found in BMS patients, which could lead to mucosal damage and heightened cytokine levels, triggering immune responses. However, despite gastric acid reflux and pepsin that may cause mucosal damage in the lower esophagus, the extension of tissue injury to oral mucosa has not been clearly demonstrated [84]. Nevertheless, it was found that GERD is one of the more common systemic comorbidities found in BMS [5, 81, 85]. These findings suggest a potential interplay between gastrointestinal symptoms and central nervous system manifestations in BMS patients. However, a comprehensive investigation into the mechanisms linking pain need to be further elucidated.

### 7.5 BMS and cognitive decline and neurological disorders

Recent studies have explored the possible connection between BMS and cognitive changes, although the evidence remains limited and not yet conclusive. For instance, research by Canfora *et al*. [86] reported impairments in specific cognitive domains, such as attention, working memory, and executive function, in patients with BMS, while constructive praxis and verbal memory remained preserved. The authors introduced the descriptive term “Burning Fog” to refer to this cognitive profile. Additionally, their study found increased Age-related White Matter Changes (ARWMC) scores in the temporal lobes of BMS patients, raising the hypothesis that microvascular brain alterations could contribute to the observed cognitive symptoms. However, a direct causal link between BMS and cerebral small vessel disease or dementia risk has not been established, and these findings should be interpreted with caution.

Another study by Dugan *et al*. [87] compared 120 individuals with BMS to 110 controls, noting significantly lower scores in verbal fluency tests among BMS patients, although no differences emerged in global cognitive function as measured by the Montreal Cognitive Assessment (MoCA). Moreover, patients with BMS and mild cognitive impairment (BMS-MCI) show greater emotional distress and poorer cognitive performance, especially in processing speed and executive function, compared to geriatric MCI patients, highlighting the role of mood and pain in cognitive decline [88].

The BMS Blueprint Persona study highlighted memory issues as a key challenge for patients, particularly impacting medication adherence. This cognitive aspect underscores the need for digital tools designed to support memory and improve treatment compliance in BMS care [89]. While there are case reports of BMS-like symptoms occurring in patients with neurodegenerative diseases, such as dementia with Lewy bodies or parkinsonism, these observations are anecdotal and do not establish a specific association between BMS and neurodegeneration. For example, a recent review by Guru *et al*. [90] found no direct evidence linking Parkinson’s disease and BMS.

An interesting, though still not fully understood, observation is the association between BMS and Restless Legs Syndrome (RLS), with both conditions potentially involving dopaminergic dysfunction. However, whether this reflects a common pathophysiological mechanism remains to be clarified, as genetic studies and mechanistic data are still inconclusive [91].

Overall, while some studies suggest that a subset of BMS patients may exhibit subtle cognitive or sensorimotor changes, these findings are not specific to BMS and should be interpreted within the broader context of chronic pain, comorbid mood disorders, and possible age-related factors. At present, no causal or diagnostic link between BMS and neurodegenerative disorders has been established. Further well-designed, longitudinal studies are needed to better understand these potential associations. Interdisciplinary collaboration between dentistry, neurology, and psychiatry remains essential in the comprehensive management of BMS patients.

### 7.6 BMS in the COVID-19 pandemic

The emergence of the COVID-19 pandemic, caused by the SARS-CoV-2 virus, has introduced new complexities in understanding BMS. COVID-19 is known to affect various bodily systems and has the potential to lead to neurological symptoms, including those related to taste disturbance and burning sensation [92, 93]. Although some COVID-19 patients have reported these symptoms, establishing a direct link between the virus and BMS continues to be a subject of ongoing research. Nevertheless, it is known that the COVID-19 pandemic has notably impacted the mental and overall health of individuals, leading to increased psychological stress and limited access to healthcare services [94]. This is particularly relevant for BMS, whose symptoms can intensify under psychological stress. The constrained access to healthcare during the pandemic may have resulted in delayed diagnosis and treatment of BMS, potentially exacerbating the condition. Indeed, Candela *et al*. [95] reported an increase in A, worsened sleep quality, and heightened pain intensity in BMS patients. Furthermore, a study by Ottaviani *et al*. [96] involving 100 BMS patients and 100 healthy controls from five Italian centers highlighted significant deep loneliness in BMS patients. This loneliness correlated with factors like age, higher education, stress, lower satisfaction in relationships, and perceived social support [96]. Moreover, during the pandemic, BMS patients exhibited increased post-traumatic stress symptoms, particularly intrusive thoughts, compared to healthy controls. These patients also showed lower levels of resilience and post-traumatic growth, indicating a reduced capacity to find positive meaning and personal growth following stressful life events [96]. This suggests that BMS patients may lack adaptive coping strategies and a positive mindset, making them more vulnerable to the negative effects of future traumas. Overall, comorbidities such as mood disorders (A, D, sleep disorders) and cardiovascular risk factors (hypertension, hypercholesterolemia, hyperhomocysteinemia) alongside the emerging impact of SARS-CoV-2 infection significantly contribute to brain aging in BMS patients. These factors, particularly cardiovascular risks, lead to microvascular injury, endothelial damage, and reduced cerebral blood flow, resulting in neuroinflammation, WMHs, and cerebral atrophy. These brain alterations may further increase chronic pain, creating a detrimental cycle. Chronic pain and WMHs serve as biomarkers of brain frailty, promoting progressive brain aging, reducing neuroplasticity, and exacerbating pain perception and mood disorders (Fig. 5).

**Fig. 5.** Conceptual model of the interaction between comorbidities, brain aging, and central mechanisms of pain chronification in Burning Mouth Syndrome (BMS). This figure illustrates potential pathways linking common comorbidities—including cardiovascular risk factors (hypertension, hypercholesterolemia, hyperhomocysteinemia), mood and sleep disorders, and the emerging effects of SARS-CoV-2 infection—to functional and microstructural brain changes. The resulting cycle of pain and brain vulnerability may further exacerbate neuroplasticity decline and mood disturbances, promoting progressive brain aging. HTN: Hypertension; HCh: Hypercholesterolemia; HHCys: Hyperhomocysteinemia; WMHs: White Matter Hyperintensities.

## 8. Classification system of ICOP 2020; ICHD-3, ICD-11, and DSM-V

In the last years, the conceptualization and categorization of BMS have undergone significant changes shaped by its clinical manifestations and treatment outcomes. Multiple classification systems have been introduced to refine its definition and differentiate it from oral burning sensations linked to various local or systemic factors. These classification systems have been developed not only for accurate disease identification but also to enhance patient understanding and acceptance of their condition [77]. Establishing universally accepted diagnostic standards has been crucial for clinicians for precise disease identification and treatment strategies and for patients to benefit from a clear diagnosis framed within a globally recognized system, aiding in their understanding and acceptance of the disease.

Although there is no consensus regarding the use of a universal and unique classification for BMS, the most used classifications are:

- The International Classification Headache Society (ICHD-3) [97];

- The International Classification of Orofacial Pain (ICOP-2020) [1];

- The International Association for the Study of Pain (IASP) for the International Classification of Diseases (ICD-11) [7] (Table 2, Fig. 6).

**Table 2.** Classification system of ICOP 2020; ICHD-3 beta version, IASP for ICD-11.

Classification	Categorization	Code and Subtypes	Pain characteristics	Exclusion criteria	Diagnostic test
ICOP 2020	Idiopathic orofacial pain	(6.1.1) BMS without somatosensory changes (6.1.2) BMS with somatosensory changes (6.1.3) Probable BMS	- Burning quality - Bilateral or unilateral - Poorly localized - Persistent - Felt superficially in the oral mucosa - Additional symptoms (xerostomia, taste disturbance)	Clinical neurological deficit - Other oral diseases - Systemic diseases (diabetes, nutritional deficiencies, thyroid disease) - medications	Quantitative somatosensory tests Blood tests (nutritional deficiencies, glycemic control, and thyroid function) oral cultures Imaging Psychometric tests
ICHD-3 beta version	Painful Cranial Neuropathies and Other Facial Pains	13.11	- Burning quality - Bilateral and diffuse - Felt superficially in the oral mucosa - Additional symptoms (xerostomia, taste disturbance)	- Clinical neurological deficit - Other oral diseases - Systemic diseases (diabetes, nutritional deficiencies, thyroid diseases) - medications	Not strictly reported Blood tests (nutritional deficiencies, glycemic control, and thyroid function)
IASP for ICD-11	Sensory disturbances affecting the orofacial complex	DA0F.0 + XS7G: Psychosocial factors present + XS8B: No psychosocial factors present + XS5B: No pain XS5D: Mild pain XS9Q: Moderate pain XS2E: Severe pain + XS1J: No distress XS3R: Mild distress XS7C: Moderate distress XS7N: Severe distress + XS71: No pain-related interference XS5R: Mild pain-related interference XS2L: Moderate pain-related interference XS2U: Severe pain-related interference	-Burning quality Associated with - emotional distress (anxiety, anger/frustration or depressed mood) - interference with orofacial functions such as eating, yawning, speaking, *etc.*	- Clinical neurological deficit - Other oral diseases - Systemic diseases (diabetes, nutritional deficiencies, thyroid diseases) - medications	Not strictly reported Psychometric tests

*A diagnosis of 6.1 Burning mouth syndrome implies that quantitative sensory testing has not been performed. 6.1.1 Burning mouth syndrome without somatosensory changes or 6.1.2 Burning mouth syndrome with somatosensory changes should be diagnosed only when quantitative sensory testing have been performed. A diagnosis of 6.1.3 is considered when pain is present for <3 months. Once 3 months have elapsed, the diagnosis becomes 6.1. ICOP: International Classification of Orofacial Pain; ICHD-3: International Classification Headache Society; IASP: International Association for the Study of Pain; ICD-11: International Classification of Diseases-11; BMS: Burning Mouth Syndrome.

**Fig. 6.** Classification and coding of burning mouth syndrome. The most used classifications for BMS with relative codes include: International Classification Headache Society (ICHD-3 beta version); International Classification of Orofacial Pain (ICOP-2020); International Association for the Study of Pain (IASP) for the International Classification of Diseases (ICD-11). BMS: Burning Mouth Syndrome; DSM-V: Diagnostic and Statistical Manual of Mental Disorders (5th Edition).

Each offers distinct perspectives on categorizing BMS based on clinical evidence and research advancements. Moreover, the ongoing updates to these classifications, readily available online, permit clinicians to evaluate changes across time.

The ICHD-3 includes BMS under the classification of “Painful Cranial Neuropathies and Other Facial Pains”. This classification defines BMS as intraoral burning or dysaesthetic sensation, recurring daily for >2 hours/day for >3 months, without clinically evident causative lesions. The pain is generally burning in character and felt superficially in the oral mucosa but the mucosa appears normal at clinical examination, and sensory testing is normal. The pain is usually bilateral; the most common site is the tip of the tongue, and it may be associated with subjective dryness of the mouth, dysaesthesia, and altered taste. Laboratory investigations and brain imaging may indicate changes in central and peripheral nervous systems. Whether secondary BMS attributed to a local (candidiasis, lichen planus, hyposalivation) or systemic disorder (medication-induced, anemia, deficiencies of vitamin B12 or folic acid, Sjogren’s syndrome, diabetes) should be considered as an entity [98].

The ICOP 2020 includes BMS in idiopathic orofacial pain and offers a more detailed and inclusive classification system for orofacial pain, aimed at improving diagnosis, treatment, and research. Differently from ICHD-3, ICOP 2020 recommends conducting somatosensory assessments to further classify BMS into subgroups based on the presence or absence of somatosensory alterations. QST is often abnormal (differentiating the two subtypes), whereas clinical sensory examination very rarely reveals slight sensory deficits however, QST currently has no definitive diagnostic value in BMS. Similarly, to ICHD-3, BMS is defined as an intraoral burning or dysaesthetic sensation, recurring daily for >2 hours/day for >3 months, without evident causative lesions on clinical examination and investigation. The pain primarily affects the tongue but may also involve other areas of the oral mucosa, such as the lips, gums, and palatal regions. Although in most patients burning is usually bilateral, on rare occasions, it is unilateral differently from ICHD-3 where all patients who reported unilateral symptoms are included in atypical facial pain. Pain/burning is associated with additional oral symptoms in two-thirds of cases such as xerostomia, dysaesthesia and altered taste. BMS is diagnosed only when all local and systemic causes have been excluded (hence, previously, primary BMS). The diagnosis of BMS in the ICOP 2020 framework requires a thorough patient history, clinical examination, and the exclusion of other possible causes of the symptoms through appropriate investigations. These may include blood tests to check for nutritional deficiencies, glycemic control, and thyroid function, as well as oral cultures and possibly imaging studies. The ICOP 2020 may also consider the impact of the disease on the patient’s quality of life and functional status and the role of psychological comorbidities frequently associated to pain, differently from ICHD-3. Moreover, this classification underscores the importance of a multidisciplinary approach to diagnosing and managing a complex condition such as BMS, which may require contributions from various healthcare professionals, including dentists, oral medicine specialists, neurologists, and psychologists, to achieve optimal patient care [1].

The IASP has contributed to the development of pain-related diagnoses in the International Classification of Diseases, 11th Revision (ICD-11), which was adopted by the World Health Organization (WHO). The ICD-11 provides a modern standard for identifying health trends and statistics globally and contains significant updates to the classification and criteria of various conditions, including chronic pain conditions like BMS. One of the pivotal advancements in ICD-11 is its refined approach to chronic pain, now recognized both as a symptom and as a distinct disease entity, complete with a supportive coding framework. A groundbreaking feature of ICD-11 is the concept of “multiple parenting”, which allows diagnostic entities to be classified under more than one category. This multifaceted approach aligns more closely with the interdisciplinary nature of medical practice, bridging gaps between specialties such as oral medicine and neurology.

According to ICD-11, BMS is classified under secondary chronic headaches (MG30.62) and more specifically coded as DA0F.0, “sensory disturbances involving the orofacial region” (Foundation ID: 618998878 in the ICD-11 browser). In this classification, BMS is characterized by an intraoral burning or dysaesthetic sensation that recurs for more than two hours per day on 50% of the days over more than three months, without evident causative lesions on clinical investigation and examination. It is characterized by significant emotional distress (A, anger/frustration, or depressed mood) or interference with orofacial functions such as eating, yawning, speaking, *etc*. The diagnosis is appropriate independently of identified biological or psychological contributors unless another diagnosis would better account for the presenting symptoms. It is possible to include additional codes that specify the intensity of pain/burning; the presence of psychosocial factors and the interference of pain [7].

The analysis of ICOP 2020, ICHD-3, and ICD-11 underscores the critical role of psychosocial components. Consequently, many authors have advocated for the integration of these classifications with the Diagnostic and Statistical Manual of Mental Disorders, Fifth Edition (DSM-5). This integration is particularly relevant for BMS within the framework of Somatic Symptom Disorder (SSD), as classified in the DSM-5 [99]. The DSM-5 code for SSD, 300.82 (F45.1), relates to conditions where individuals experience an intense focus on physical symptoms. These symptoms, such as the chronic burning pain characteristic of BMS, lead to substantial health-related A, disproportionate concerns about the symptoms, and significant distress or functional impairment. In the case of BMS, the emphasis on the subjective experience of distress and the extensive psychological impact, including health-related A and concern over symptoms, aligns with the SSD criteria. The persistent nature of BMS symptoms can perpetuate a cycle of distress and functional impairment, reflecting the SSD framework [99]. This alignment highlights the importance of a holistic approach to diagnosing and treating BMS. Such an approach should address not only the physical symptoms but also the psychological and social dimensions of the condition, emphasizing the integral role of psychosocial factors in the experience and management of BMS.

## 9. Diagnosis of BMS

The diagnosis of BMS presents a significant clinical challenge, as it requires the exclusion of other systemic or local disorders that could contribute to oral cavity pain. To accurately diagnose and treat this condition, clinicians should consider patients’ characteristics and pain characteristics at the same time [17].

Firstly, a detailed examination of the oral cavity, and extensive analysis of socio-demographic characteristics, habits, body mass index (BMI), and medical history including current medications, any history of mood disorders, systemic diseases, and familiarity with psychiatric and cognitive impairment is required for every patient (Table 3).

**Table 3.** Characteristic assessment of BMS patients’ profile.

Category	Items
Characteristics of the patient	Gender
	Age
	Education level
	Marital status
	Job status
	BMI (with categories):
	- Underweight: BMI: <18.5
	- Normal weight: BMI: 18.5–24.9
	- Overweight: BMI: 25–29.9
	- Obesity class I: BMI: 30–34.4
	- Obesity class II: BMI: 35–39.9
	- Obesity class III: BMI: 40 or greater
Socio-demographic Characteristics	Smoking
	Alcohol consumption
	Physical activity
Medical Comorbidities	Hypertension
	Hypercholesterolemia
	Previous Myocardial infarction
	Other Cardiovascular diseases
	Hyperhomocysteinemia
	Asthma
	Gastroesophageal reflux disease (GERD)
	Endocrine diseases
	Benign Prostatic Hypertrophy
	Hypothyroidism
	Hyperthyroidism
	HCV infection
	HBV infection
	Neoplastic disease
	Others
Drugs Intake	ACE-inhibitors
	Calcium Channel blockers
	Angiotensin II receptor antagonists (ARBs)
	Thiazide Diuretics
	Beta-blockers
	Statins
	Ezetimibe
	Antiplatelets
	Blood thinners
	Bisphosphonates or antiresorptive drugs
	Levothyroxine sodium
	Steroids
	Proton Pump inhibitors
	Others

*Recommended assessment for all BMS patients, to identify potential comorbidities and contributing factors (socio-demographic data, habits, medical conditions, and medications). BMI categories follow WHO criteria. BMI: body mass index; ACE: angiotensin-converting enzyme; HCV: Hepatitis C Virus; HBV: Hepatitis B Virus.*

An extensive clinical examination should investigate signs of parafunctional habits, erythema, irritation, glossitis, atrophy of oral mucosa or tongue, mucosal ulceration or other abnormalities.

Medical history of the patients and laboratory investigation are also essential to exclude any possible local and systemic conditions causing oral burning [1]. For instance, there is a range of medications acting as potential triggers for BMS-like symptoms, such as antihypertensive drugs, particularly angiotensin-converting enzyme (ACE) inhibitors or antiretrovirals [100, 101]. It is possible to perform challenge, dechallenge, and rechallenge to determine if a medication has caused the burning sensation. This procedure is useful for establishing a causal relationship between the intake of a specific medication and the onset of burning symptoms, providing a structured methodology for identifying pharmacological triggers. A thorough medical history is necessary to assess if the burning sensation started after the introduction of a new medication, and if so, to proceed with this methodology. A detailed analysis of the pain’s characteristics—such as its onset, duration, location, and any factors that may exacerbate or alleviate it—and the presence of extraoral symptoms (medically unexplained extraoral physical symptoms) is crucial. This analysis should employ both qualitative and quantitative pain assessment tools (Table 4).

**Table 4.** Characteristic assessment of pain in BMS patients.

Characteristic	Items
Site	Diffuse across oral mucosa.
	Bilateral or unilateral
	Localized on tongue or other oral mucosa sites
Onset	Spontaneous or triggered burning/pain
Triggers	Dental treatment
	Stressful life events
	New medications
	Illness
	COVID-19 or vaccines
Character	Burning, Tender, Hot, Stinging, Scalding, Numbness, Discomfort, Raw, Unpleasant, Annoying
	Superficial oral mucosa pain
Intensity	Moderate, Severe
Time/Duration	Pain/burning >2 hours a day.
	Occurs more than 3 months
Pattern of Pain	Continuous, Intermittent
	Same morning/afternoon/evening
	Worst in afternoon/evening
	Worst in morning
Exacerbating Factors	Eating, chewing, or drinking
Relieving Factors	Eating, contact with certain foods or drinks
Additional Oral Symptoms	Xerostomia
	Dysgeusia
	Intraoral Foreign Body Sensation or cenesthopathy
	Globus Pharyngeus
	Discerptions (color alterations: white tongue, red areas bullae and morphological alterations: swellings, dysmorphisms, teeth enlargements)
	Sialorrhea
	Itching
	Tingling sensation
	Occlusal Dysesthesia
	Oral dyskinesia
	Dysosmia
	Subjective Halitosis
Psychological comorbidity and Feelings that amplify symptomatology	Anxiety
	Depression
	Sleep disturbance (Trouble falling or staying asleep, or sleeping too much)
	Loneliness
	Stress
	Personality disorders
	Obsessive-compulsive disorder (OCD)
	Hypochondria (Worries about complaints)
	Not being able to stop or control worrying
	Lack of time/work-related stress/caring responsibilities/finances
	Lack of support/interpersonal conflicts
	Different opinions from caregivers/not been taken seriously
	Little interest or pleasure in doing things
	Feeling down, depressed, or hopeless
Medically Unexplained Extraoral Symptoms	Irritable bowel syndrome
	Fibromyalgia
	Chronic fatigue syndrome
	Tinnitus
	Ophthalmodynia
	Skin burning/itching
	Vulvodynia
	Functional dyspepsia
	Tensive Headache
	Dizziness
	Restless legs syndrome
	Ear itching
	Nasal itching/Burning
	Low Back pain
	Myofascial pain
	Anal itching
	Asthenia
	Premature Ejaculation

*This table summarizes key characteristics of pain and associated symptoms in Burning Mouth Syndrome (BMS) patients. It is intended to guide comprehensive clinical assessment, helping to document pain features, triggers, time course, exacerbating/relieving factors, additional oral and extraoral symptoms, and psychological comorbidities. A multidimensional evaluation of these aspects supports accurate diagnosis and personalized management.*

The quality of pain is often described as burning, tender, tingling, hot, stinging, scalding, numbness, discomfort, raw, unpleasant, or annoying. The intensity of pain varies from mild to severe. BMS presents as a predominantly burning, neuropathic pain with a diurnal increase in intensity, and while initially challenging, it can be successfully managed in most cases through a collaborative, empathetic approach involving the other medical colleagues [29, 102]. BMS patients generally report oral burning/pain that diffuse to the entire oral mucosa or restricted to the tongue, but it can also affect buccal mucosa, lip, floor of the mouth, gingiva, and palate or rarely burning may be unilateral. When pain is unilateral, it is essential to differentiate BMS from other orofacial pain conditions, such as persistent idiopathic facial pain (PIFP), persistent idiopathic dentoalveolar pain (PIDAP), and trigeminal neuralgia (TN) (Table 5, Ref. [1]).

**Table 5.** Differential diagnosis of BMS from other chronic oral pain conditions based on ICOP 1st edition, 2020 [1].

Condition	Quality of Pain	Pain Intensity	Location	Diagnostic criteria
Burning Mouth Syndrome (BMS)	Burning, tender, tingling, hot, stinging, scalding, numbness, discomfort, raw, unpleasant, or annoying	Mild to severe	Diffuse to the entire oral mucosa or restricted to the tongue; can affect buccal mucosa, lip, floor of the mouth, gingiva, and palate; rarely unilateral	(A) Oral pain fulfilling criteria B and C (B) Recurring daily for >2 h per day for >3 mon (C) Pain has both of the following characteristics: (1) burning quality (2) felt superficially in the oral mucosa (D) Oral mucosa is of normal appearance, and local or systemic causes have been excluded
Trigeminal Neuralgia (TN)	Sharp, stabbing, electric shock-like, abrupt in onset and termination	Severe	Unilateral, usually affecting one side of the face, limited to the distribution of one or more divisions of the trigeminal nerve and triggered by innocuous stimuli	(A) Recurrent paroxysms of unilateral facial pain in the distribution(s) of one or more divisions of the trigeminal nerve, with no radiation beyond, and fulfilling criteria B and C (B) The pain has all the following characteristics: (1) lasting from a fraction of a second to 2 minutes (2) severe intensity (3) electric shock-like, shooting, stabbing or sharp in quality (C) Precipitated by innocuous stimuli within the affected trigeminal distribution
Persistent Idiopathic Facial Pain (PIFP)	Dull, aching, or burning	Mild to severe	Unilateral or bilateral, often vague and poorly localized	(A) Facial pain fulfilling criteria B and C: (B) Recurring daily for >2 h/d for >3 mon (C) Pain has both of the following characteristics: (1) poorly localized, and not following the distribution of a peripheral nerve (2) dull, aching or nagging quality (D) Clinical and radiographic examinations are normal, and local causes have been excluded
Persistent Dentoalveolar Pain (PIDAP)	Persistent, aching, sometimes throbbing	Mild to moderate	Localized to the teeth or alveolar processes	(A) Intraoral dentoalveolar pain fulfilling criteria B and C (B) Recurring daily for >2 h/d for >3 mon (C) Pain has both of the following characteristics: (1) localized to a dentoalveolar site (tooth or alveolar bone) (2) deep, dull, pressure-like quality (D) Clinical and radiographic examinations are normal, and local causes have been excluded

The early onset of the disease is characterized by a less severe symptomatology in the morning, getting worse during the day, although generally alleviated by eating and drinking, but in a long-standing disease these criteria may be not respected [1]. Frequently, most patients report additional symptoms, such as xerostomia, alteration in taste perception, phantom taste, alteration in sensory perception, globus, and others that may differ between patients, resulting in a varying clinical presentation [5, 10, 36]. The characteristics of symptomatology are reported in Table 4. The additional symptoms frequently complicate the disease and contribute to diagnostic delay because many instrumental exams and evaluations by other specialists are required to exclude possible related local and systemic conditions with the same symptoms. Therefore, BMS may remain under-recognized and under-appreciated by both dental and medical professionals for a long time. It took an average of 30 months from the onset of symptoms until a definitive diagnosis of BMS was achieved and every patient consulted almost three specialists, receiving several misdiagnoses before a proper diagnosis [5]. Additionally, the somatosensory screening tests may be considered to assess sensory nerve dysfunction in BMS patients. A thorough assessment should also include the metabolic profile evaluation through hematological tests, to rule out the nutritional, hormonal, autoimmune, and thrombophilia conditions that may underlie or contribute to symptoms. The laboratory tests required in BMS patients are available in (Table 6).

**Table 6.** Laboratory investigations in BMS patients.

Category	Blood Tests
Complete Blood Count (CBC)	White Blood Count (WBC), Red Blood Count (RBC), Platelet Count (Plt), Hemoglobin, Hematocrit, Mean corpuscular volume (MCV)
Glucose Metabolism	Glucose, Glycated hemoglobin (Hemoglobin A1C)
Kidney Function	Blood urea, Creatinine
Lipid Profile	Total cholesterol, High-density lipoprotein (HDL), Triglycerides, Low-density lipoprotein (LDL)
Iron Studies	Serum Iron, Serum Ferritin, Transferrin
Electrolytes	Sodium, Potassium, Magnesium
Liver Function Tests	Aspartate Phosphatase (AST or SGOT), Alanine Aminotransferase (ALT or SGPT), Alkaline Phosphatase (ALP), Total Bilirubin, Direct Bilirubin, Indirect Bilirubin, Albumin
Protein Studies	Protein electrophoresis serum test
Thyroid Panel	Thyroid stimulating hormone (TSH), Free T3 and T4 thyroid levels
Immune Panel	Antinuclear Antibodies (ANA), Extractable Nuclear Antigen Antibodies (ENA; anti-SSA and anti-SSB), C-reactive protein (CRP) test
Hormonal Tests	Prolactin, oestradiol, testosterone
Nutritional Deficiency/Vitamin Levels	Vitamin B12, B1, B6 test, Vitamin D test, Zinc
Urine Test	Routine Urinalysis
Coagulation Profile	PT, PTT & INR, Fibrinogen
Other Specific Test
	Homocysteine, Folate
	NSE (Neuron Specific Enolase)
Hypercoagulable Workup	Protein C activity, Protein S activity Plasma antithrombin III; Anti-β2-glycoprotein I antibodies (antiβ2GPI; IgM and IgG); Anticardiolipin antibodies (ACA; IgM and IgG); Lupus anticoagulants (Las)
Genetic tests to assess for thrombophilia	*MTHFR C677T*: To check for a mutation that might lead to hyperhomocysteinemia, which could increase the risk of clotting disorders. Fattore V Leiden (Factor V Leiden): To detect a specific gene mutation that leads to a higher chance of forming abnormal blood clots. Fattore II or Prothrombin *G20210A*: To identify a mutation in the prothrombin gene that can lead to an increased risk of thrombosis. *MTHFR A1298C*: Similar to the *C677T* mutation, to check for another common variant that may affect homocysteine levels and potentially lead to clotting issues. *PAI 1 GG*/*5G*: To identify polymorphisms in the plasminogen activator inhibitor-1 (*PAI-1*) gene that could be linked to thrombotic disorders. Factor V *Y1702*: This appears to be a less common genetic test and may refer to a specific mutation within the Factor V gene related to clotting risk, similar to Factor V Leiden. Factor V *HR2 (H1299R)*: A test for another mutation in the Factor V gene, which can be associated with an increased risk of venous thromboembolism.

*AST or SGOT: Aspartate Aminotransferase or Serum Glutamic-Oxaloacetic Transaminase; ALT or SGPT: Alanine Aminotransferase or Serum Glutamic-Pyruvic Transaminase; anti-SSA: Anti-Sjögren’s-syndrome-related antigen A (Ro) antibody; anti-SSB: Anti-Sjögren’s-syndrome-related antigen B (La) antibody; PT: Prothrombin Time; PTT: Partial Thromboplastin Time; INR: International Normalized Ratio; ACA: Anticardiolipin Antibody; IgM: Immunoglobulin M; IgG: Immunoglobulin G; MTHFR: Methylenetetrahydrofolate Reductase.*

Comprehensive diagnostic screening for nutritional deficiencies and anemia is imperative because deficits in ferritin, iron, vitamin B12, folate, and zinc are often present in iron-deficiency anemia and vitamin B12-deficiency anemia. These conditions can lead to a secondary form of oral burning. Therefore, when deficiencies are identified, targeted treatment to correct them is crucial. This typically involves supplementation and dietary modifications to replenish the deficient micronutrients [92]. Upon correcting these deficiencies, a careful reassessment of the patient’s symptoms is necessary to determine whether the oral burning has resolved or improved. This step is important as it can help differentiate between a secondary burning related to nutritional deficits and BMS, where the symptoms may not respond to nutritional supplementation.

Similarly, hyperhomocysteinemia is a condition frequently observed in patients with BMS. It is often associated with folate deficiency, as well as vitamins B6 and B12, all of which are necessary for homocysteine metabolism [103]. Management of hyperhomocysteinemia includes supplementation with these vitamins, and it’s critical to monitor the patient’s response to treatment because hyperhomocysteinemia is a risk factor for various neurodegenerative and vascular brain pathologies, including increased stroke risk, cognitive decline, dementia, and mood disorders through mechanisms such as oxidative stress, endothelial dysfunction, excitotoxicity, and impaired methylation processes [104, 105, 106, 107]. Specifically, testing for mutations in the Methylenetetrahydrofolate Reductase (MTHFR) gene (C677T and A1298C) is advised in patients with hyperhomocysteinemia exhibiting suboptimal responses to vitamin B supplementation. Such mutations can affect the metabolism of homocysteine and, by extension, influence the patient’s response to treatment.

Additionally, when an MRI of the brain reveals a high burden of WMHs that cannot be accounted for by common risk factors, such as hypertension or aging, genetic testing may uncover a predisposition to vascular disorders that impair cerebral blood flow. This is particularly relevant in patients with a history of deep vein thrombosis, pulmonary embolism, pre-eclampsia, or recurrent miscarriages, where an underlying thrombophilic condition might exist.

In all cases, genetic tests should be considered as part of a comprehensive evaluation that includes a detailed clinical examination, patient’s medical history, and if necessary, genetic counseling. It is crucial to interpret the results within the overall clinical context of the patient and not in isolation. A Comprehensive Evaluation of Coagulation Parameters in the context of BMS is meticulously detailed in the Tables 6 and 7, with a particular focus on the modifications of test results in patients who are undergoing treatment with antiplatelet agents and blood thinners, which are commonly prescribed for individuals with BMS.

**Table 7.** Comprehensive evaluation of coagulation parameters in the context of BMS: detailed insights and therapeutic considerations.

Test	High Value mean	Low Value mean	Effects of Antiplatelets	Effects of Blood Tinners
Prothrombin Time (PT)	Indicates blood clotting is slower than normal, potential bleeding disorder or anticoagulant use	Indicates blood clotting is faster than normal, potential risk of thrombosis	No direct effect	Prolonged PT is expected with warfarin therapy
Activated Partial Thromboplastin Time (aPTT)	Indicates blood takes longer to clot, may suggest bleeding disorder, factor deficiency, or anticoagulant use	Not typically clinically significant	No direct effect	Prolonged aPTT may occur with heparin therapy
Fibrinogen	Indicates acute phase reactant response, may suggest inflammation, infection, or tissue injury	Possible risk of bleeding disorders or fibrinolytic states	No direct effect	No direct effect
Homocysteine	Increased risk of cardiovascular disease and thrombosis	Not typically clinically significant	No direct effect	No direct effect
Folate	Generally not clinically significant, but can indicate supplementation or dietary intake	Possible deficiency, risk of anemia, other hematological disorders, and associated with hyperhomocysteinemia	No direct effect	No direct effect
Protein C Activity	Increased risk of bleeding (rarely observed)	Increased risk of thrombosis	No direct effect	No direct effect
Protein S Activity	Increased risk of bleeding (rarely observed)	Increased risk of thrombosis	No direct effect	No direct effect
Antithrombin III	Possible non-specific increase, not typically clinically significant	Increased risk of thrombosis	No direct effect	No direct effect
Anti-β2-glycoprotein I Antibodies (IgM and IgG)	May indicate an autoimmune condition such as antiphospholipid syndrome	Negative or low values typically do not indicate an increased risk of thrombosis	No direct effect	No direct effect
Anticardiolipin Antibodies (IgM and IgG)	May indicate an autoimmune condition such as antiphospholipid syndrome	Negative or low values typically do not indicate an increased risk of thrombosis	No direct effect	No direct effect
Lupus Anticoagulants	May indicate an autoimmune condition such as antiphospholipid syndrome or other coagulation disorders	Negative or low values typically do not indicate an increased risk of autoimmune conditions	No direct effect	May interfere with test, especially in patients on warfarin or heparin

*This panel of coagulation tests is proposed to identify potential prothrombotic states or autoimmune abnormalities (e.g., antiphospholipid antibodies), which may contribute to microvascular alterations hypothesized in the pathogenesis of Burning Mouth Syndrome (BMS). Their use is intended to guide individualized diagnostic workup, particularly in patients with atypical clinical features or risk factors. IgM: Immunoglobulin M; IgG: Immunoglobulin G.*

Recently, Kishore and collaborators have proposed using NSE as a novel biomarker for peripheral neuropathies. Found in neurons and neuroendocrine cells, NSE plays a crucial role in glycolysis. Its utility in clinical diagnostics lies in its ability to indicate neuronal damage, making it a valuable tool for assessing various neuropathic conditions. Incorporating NSE measurement into the screening process for peripheral neuropathies allows clinicians to detect early signs of neuronal damage, thereby facilitating timely intervention and management [47].

Clinicians should perform an electrocardiogram (ECG) before starting pharmacological treatment, especially when prescribing psychotropic drugs. The ECG helps to identify cardiovascular risks, such as prolonged QT interval, conduction disorders, or cardiac ischemia, which may affect treatment decisions. QTc prolongation (normal: <430 ms in men, <450 ms in women; borderline: 430–450 ms in men, 450–470 ms in women; prolonged: >450 ms in men, >470 ms in women) is often drug-induced and is a known risk factor for torsades de pointes, a potentially fatal arrhythmia [108].

Many psychiatric drugs, including some antipsychotics and antidepressants, are known to potentially cause QT interval prolongation. A baseline ECG can establish whether the patient has a prolonged QT interval before treatment initiation, allowing the physician to make informed decisions regarding the prescription. The incidence of this adverse event is unpredictable, but a common observation is that most patients have at least one identifiable risk factor in addition to antidepressants exposure, such as female gender, age older than 60 years, hypokalemia, and a previous diagnosis of cardiovascular disease [109]. Therefore, the Food and Drug Administration recommends evaluating periodic ECG monitoring in all patients who begin treatment with these drugs with monitoring of the electrolytes, especially potassium [110]. Following this initial assessment, imaging techniques, particularly MRI of the brain, the brain stem, and the maxillofacial area should be employed as supplementary tools. MRI of the brain and brain stem can help identify any central nervous system pathology, such as WMHs [36] or space-occupying lesions [111] which are vital for a comprehensive understanding of the patient’s condition.

For cases presenting with unilateral and localized burning sensations on the gingiva, orthopantomography (OPT) and Cone Beam Computed Tomography (CBCT) of the jaw are recommended. These imaging modalities are essential for ruling out dental causes of pain, including local infections of the jaw, which might mimic or exacerbate BMS symptoms. Further, in the assessment of BMS, particularly when patients report symptoms of dry mouth, it is crucial to include comprehensive immunological testing as part of the diagnostic workup. The assessment should encompass Antinuclear Antibodies (ANA), Extractable Nuclear Antigen Antibodies (ENA; anti-SSA: Anti-Sjögren’s-syndrome-related antigen A; anti-SSB: Anti-Sjögren’s-syndrome-related antigen B), and C-reactive protein (CRP) levels. Elevated levels of these markers can be instrumental in unveiling underlying immunological conditions, notably Sjogren’s Syndrome. Routine ANA testing is not recommended in BMS patients without xerostomia or other clinical signs suggestive of systemic autoimmune disease. Targeted ANA testing may be considered in selected patients with additional risk factors or symptoms [112]. The presence of these antibodies, alongside raised CRP levels, points to an autoimmune process that may be contributing to the patient’s symptoms, necessitating further evaluation. Imaging studies of the salivary glands, such as sialography, scintigraphy, salivary gland ultrasonography, or MRI of the salivary glands, become essential in this context. These imaging modalities offer valuable insights into glandular structure and functionality, helping to distinguish between objective xerostomia—where there is a quantifiable decrease in salivary flow—and subjective xerostomia, where patients perceive dryness without a measurable reduction in saliva production [113, 114].

In patients that report globus or exhibit erythematous lesions of the soft palate or dental erosions, these may indicate an underlying GERD. In such cases, a consultation with a gastroenterologist is mandatory to address and manage the potential reflux contributing to BMS [81, 85]. By adhering to this structured diagnostic approach, healthcare professionals can ensure a comprehensive evaluation of BMS, addressing both the primary symptoms and any underlying or contributing conditions. This meticulous attention to detail ensures that treatment strategies are well-informed and tailored to the individual needs of the patient, thereby improving outcomes and patient satisfaction.

## 10. Multidimensional clinical assessment of BMS: insights from IMMPACT

In the early 2000s, the international pain research community identified a critical gap: the lack of standardized outcome measures in chronic pain studies. This led to the establishment of the Initiative on Methods, Measurement, and Pain Assessment in Clinical Trials (IMMPACT) [115], which introduced a set of core outcome domains designed to capture the multidimensional nature of pain—including its intensity, as well as its physical, emotional, and social impacts. While IMMPACT recommendations were originally developed for clinical trials, they can also guide and enrich the clinical evaluation of complex pain conditions such as BMS, promoting a more comprehensive understanding of each patient’s experience and needs.

This is not intended as a recommendation to routinely administer all the listed questionnaires to every patient. Rather, it encourages clinicians to adopt a flexible, patient-centered approach, choosing appropriate tools based on clinical judgment and the patient’s presentation. Table 8 summarizes validated instruments used in research and selectively applicable in clinical settings to deepen the assessment of BMS-related impairment.

**Table 8.** Integrated assessment tools for comprehensive evaluation of pain and psychological profile in BMS patients.

Abbreviations		Questionnaires	Assessed Domains	Specific focus
Domain 1				
	VAS	Visual Analogue Scale	Pain Intensity	A 10 cm line representing pain intensity, with the left end indicating no pain and the right end indicating the worst possible pain. Patients mark their pain level on the line. Not suitable for patients with cognitive or physical impairments.
	NRS	Numeric Rating Scale	Pain Intensity	An 11-point scale for pain intensity, from 0 (no pain) to 10 (the most terrible pain imaginable). Patients verbally choose or circle the number representing their pain. Practical, simple, and requires no visuo-motor coordination.
	GCPS	Graded Chronic Pain Scale	Pain intensity	Consists of three scales that assess pain intensity from 0 to 10, estimating current pain, worst pain, and average pain intensity over the last 30 days.
Domain 2				
	PCS	Pain Catastrophizing Scale	Impact of pain and quality	To assess thoughts and feelings that contribute to the amplification of pain perception.
	BPI	Brief Pain Inventory	Impact of Pain and quality	To evaluate how pain affects various aspects of daily life and its severity.
	SF-MPQ	Short Form of McGill Pain Questionnaire	Impact of Pain and quality	To assess different qualities of pain experience (*e.g.*, throbbing, shooting, stabbing, *etc.*).
	PDQ	PainDetect Neuropathic Pain Questionnaire	Impact of Pain and quality	To identify neuropathic pain components in patients’ pain experience.
	DN4	Screening Questionnaire for Neuropathic Pain	Impact of Pain and quality	To screen neuropathic pain through symptom checklist.
	PVAQ	Pain Vigilance and Awareness Questionnaire	Impact of Pain and quality	To evaluate individual’s attention to and awareness of pain and how the patient perceive and focus on their pain.
Domain 3				
	HADS	Hospital Anxiety and Depression Scale	Emotional Impact of Pain	To evaluate levels of anxiety and depression that may accompany chronic pain conditions.
	SCL-90 R	Symptom Checklist-90-Revised	Emotional Impact of Pain	To evaluate a broad range of psychological problems and symptoms of psychopathology.
	POMS	Profile of Mood States	Emotional Impact of Pain	To assess transient, distinct mood states: Tension-Anxiety, Depression-Dejection, Anger-Hostility, Vigor-Activity, Fatigue-Inertia, and Confusion-Bewilderment.
	PHQ-9	Patient Health Questionnaire-9	Emotional Impact of Pain	To screen for depression severity, which can be impacted by chronic pain.
	PHQ-4	Patient Health Questionnaire-4	Emotional Impact of Pain	To screen for anxiety and depression.
	BAI-BDI	Beck Anxiety and Depression Inventories	Emotional Impact of Pain	To assess the severity of anxiety and depression.
	DEPS	Depression Scale	Emotional Impact of Pain	To screen for symptoms of depression.
	QIDS-SR16	16-item Quick Inventory of Depressive Symptomatology	Emotional Impact of Pain	To measure the severity of depressive symptoms.
	PASS	Pain Anxiety Symptom Scale	Emotional Impact of Pain	To assess anxiety specifically related to pain.
	POMS	Profile of Mood States	Emotional Impact of Pain	To assess transient, distinct mood states in patients with various medical conditions.
	HAM-D	Hamilton Depression Rating Scale	Emotional Impact of Pain	To assess the severity of depression.
	HAM-A	Hamilton Anxiety Rating Scale	Emotional Impact of Pain	To evaluate the severity of anxiety symptoms.
	Z-SRDS	Zung Self-Rating Depression Scale	Emotional Impact of Pain	To measure levels of depression.
	GAD-7	General Anxiety Disorder-7	Emotional Impact of Pain	To assess anxiety severity, often heightened in chronic pain conditions.
	ASI	Anxiety Sensitivity Index	Emotional Impact of Pain	To measure the extent to which individuals are concerned about anxiety symptoms.
	STAI-Y2eY2	State-Trait Anxiety Inventory	Emotional Impact of Pain	Differentiates between the temporary condition of “state anxiety” and the more general and long-standing quality of “trait anxiety”.
	GHQ-12	12-Item General Health Questionnaire	Emotional Impact of Pain General mental Health status	To screen for psychiatric disorders and assesses overall mental health.
	DASS-21	Depression Anxiety Stress Scales-21	Emotional Impact of Pain	To measure the severity of depression, anxiety, and stress highlighting emotional distress in individuals.
	IES-R-6	Impact of Event Scale-Revised	Emotional Impact of Pain Post-Traumatic Stress Symptoms	To assess symptoms of post-traumatic stress, focusing on specific aspects such as intrusive thoughts.
	PTGI-SF	Post Traumatic Growth Inventory Short Form	Emotional Impact of Pain Post-Traumatic Growth	To measure the degree of personal growth or positive psychological change following trauma.
	CDRS-10	Connor-Davidson Resilience Scale	Emotional Impact of Pain Resilience	To evaluate levels of resilience, indicating an individual’s ability to cope with and bounce back from adversity.
	ULS-8	Short-form UCLA Loneliness Scale-8	Emotional impact of pain Loneliness	To measure feelings of loneliness and social isolation.
	MSPSS	Multidimensional Scale of Perceived Social Support	Emotional impact of pain Social Support	To assess the perception of social support from family, friends, and significant others.
	SIDAS	Suicidal Ideation Attributes Scale	Emotional impact of pain Suicidal Thoughts	To measure the frequency and intensity of suicidal ideation.
	NEO PI-R	NEO Personality Inventory-Revised	Emotional impact of pain Personality	To measure five dimensions of personality (Neuroticism, Extraversion, Openness, Agreeableness, Conscientiousness) and their facets.
	MMPI	Minnesota Multiphasic Personality Inventory	Emotional impact of pain Personality	Used to identify personality and psychosocial disorders, revealing characteristics in BMS patients similar to those in chronic pain patients.
	SCID-II	Structured Clinical Interview for DSM-IV Axis II Personality Disorders	Emotional impact of pain Personality	To evaluate personality disorders and split in Cluster A Cluster B.
	EPQ-RSC	Eysenck Personality Questionnaire-Revised Short Scale for Chinese	Emotional impact of pain Personality	To evaluate mental status, and level of anxiety and depression.
	MCMQ	Medical Coping Modes Questionnaire	Emotional impact of pain Coping Mechanisms	To measure 3 cognitive-behavioral, illness-related coping strategies
	TIPI	Ten-Item Personality Inventory	Emotional Impact of pain Personality	To explore the impact of personality on pain and related experiences, highlighting neuroticism’s significant association with pain symptoms.
	OCI	Obsessive-Compulsive Disorder	Emotional Impact of pain Personality	To evaluate the symptoms of obsessive-compulsive disorder.
	C-PAS	Compulsive Personality Assesment Scale	Emotional Impact of pain Personality	To evaluate traits associated with compulsive personality disorder.
	PSQI	Pittsburgh Sleep Quality Index	Emotional Impact of Pain Sleep	To Evaluate sleep quality and disturbances, which can be affected by pain.
	ISI	Insomnia Severity Index	Emotional Impact of Pain Sleep	To Assess the severity of insomnia symptoms related to pain conditions.
	ESS	Epworth Sleepiness Scale	Emotional Impact of pain Sleep	To assess the subjective daytime sleepiness.
	SLP-6 e 9	Sleep Problem Index	Emotional Impact of pain Sleep	To assess common sleep disturbances such as trouble sleeping, nightmares and to evaluate sleep habits, such as sleep duration, difficulty waking up and daytime sleepiness.
	GOHAI	Global Oral Health Assessment Index	Impact on quality of life Oral Health Quality	To assess the impact of oral health on quality of life, focusing on functional, psychosocial, and pain/discomfort aspects.
	OHIP-14	Oral Health Impact Profile	Impact on quality of life Oral Health Impact	To Measure the social and psychological effects of oral conditions on quality of life.
	OHR-QoL	Oral Health-Related Quality of Life	Impact on quality of life Oral Health Quality	To measure how oral health affects an individual’s quality of life.
	IPQ-R	Illness Perception Questionnaire	Impact on quality of life Illness Perceptions	To evaluate how patients perceive their illness, including beliefs about causes, consequences, and control/cure.
	HRQoL	Health-Related Quality of Life	Impact on quality of life General health status	To Assess how an individual’s health status affects their quality of life.
	GP-CORE	General Population-Clinical Outcomes in Routine Evaluation	Impact on quality of life General Health status	To monitor and evaluate a range of psychological issue, including well-being and life function in the general population.
	SF-36	Short Form Health Survey 36	Impact on quality of life General Health Status	A comprehensive measure of health status covering physical, emotional, and social well-being.
	EQ-5Q	EuroQol Questionnaire	Impact on quality of life General Health Status	To measure health-related quality of life. It consists of two parts: a descriptive system and the EQ visual analogue scale (EQ VAS). The descriptive system captures five dimensions: mobility, self-care, usual activities, pain/discomfort, and anxiety/depression, each with three levels of severity.
	GH-28	General Health Questionnaire	Impact on quality of life General Mental health	To screen for psychiatric disorders and assesses overall mental health.
	PEG	3-item Pain, Enjoyment, and General activity	Impact on quality of life Pain and General Well-being	To Assess pain intensity, interference with enjoyment of life, and interference with general activity.
Domain 4				
	PGIC	Patient Global Impression of Change	Ratings of global improvement and satisfaction	To evaluate the overall change since the beginning of the treatment on a seven-point scale.
	CSQ	Client Satisfaction Questionnaire	Ratings of global improvement and satisfaction	To measure patient satisfaction with healthcare services. It typically consists of multiple items that explore the quality of care, the information provided, the provider’s behavior, and the overall healthcare experience.
	TSQM	The Treatment Satisfaction Questionnaire for Medication	Ratings of global improvement and satisfaction	To evaluate patients’ satisfaction with their medications exploring information about the patient’s experience with their treatment, focusing on several key aspects that affect their overall satisfaction and adherence to the prescribed regimen.
	CGI-S CGI-I CGI-E	Clinical global Severity of Illness Global Improvement; Efficacy Index	Ratings of global improvement and satisfaction	To evaluate the patient’s response to treatment and side effects based on a global overview of their condition.
Domain 5				
	AEQ	Adverse Events Questionnaire	Symptoms and Adverse Events associated with medical treatments	To Collect patient-reported adverse events or side effects from treatment.
	UKU SERS	UKU Side Effect Rating Scale	Symptoms and Adverse Events associated with medical treatments	To assess side effects of psychotropic medicationsIt includes a clinician-rated version and a self-rating version for patients.
	ASEC	Antidepressant Side-Effect Checklist	Symptoms and Adverse Events associated with medical treatments	To assess side effects of psychotropic medications.
	PROMISE	Patient Reported Outcome Measure, Inquiry into Side-Effects	Symptoms and Adverse Events associated with medical treatments	To evaluate and monitor symptoms and adverse events associated with medical treatments. It provides a standardized set of measures that can assess physical, mental, and social health from the patient’s perspective.
Domain 6				
	PMQ	The Pain Medication Questionnaire	Adherence of treatment and discontinuation	It specifically targets patients who are prescribed pain medications and explore patients’ beliefs about pain medication, their adherence to pain management regimens, their knowledge about their pain medication, and behaviors that might suggest misuse or abuse.
	TSQM	Treatment Satisfaction Questionnaire for Medication	Adherence of treatment and discontinuation	To understand the patient’s perspective on the medication they are receiving. This questionnaire can help to predict medication adherence, as greater satisfaction with medication is often linked to better adherence.
	BMQ	The Brief Medication Questionnaire	Adherence of treatment and discontinuation	To identify potential issues with medication adherence among patients. It’s designed to quickly gather information on a patient’s medication-taking behavior and barriers to adherence.
	MARS	Medication Adherence Report Scale	Adherence of treatment and discontinuation	To measure adherence to medication regimens. It contains a set of statements regarding medication-taking behaviors to which patients respond on a 4 or 5-point scale, with higher scores indicating better adherence.

*This is a list of validated tools for the comprehensive assessment of pain and psychological profile in BMS patients. Tools are grouped by domain: pain intensity, pain impact and quality, emotional impact, global improvement and satisfaction, treatment-related adverse events, and treatment adherence.*

IMMPACT recommends evaluating chronic pain patients across six core domains: pain intensity, physical functioning, emotional well-being, participant ratings of improvement and satisfaction with treatment, adverse events, and participant disposition (including adherence and reasons for treatment withdrawal). This framework is particularly relevant to BMS, where symptoms extend beyond pain intensity and may affect multiple aspects of life.

Suggested tools for each domain include:

• Pain Intensity (Domain 1): Numerical Rating Scale (NRS), Visual Analog Scale (VAS).

• Physical Functioning (Domain 2): Multidimensional Pain Inventory (MPI), Brief Pain Inventory (BPI), Pain Disability Index (PDI), which assess the impact of pain on activity, mood, relationships, sleep, and quality of life.

• Emotional Functioning (Domain 3): Hospital Anxiety and Depression Scale (HADS), Beck Depression Inventory (BDI), Profile of Mood States (POMS).

• Global Improvement and Satisfaction (Domain 4): Patient Global Impression of Change (PGIC).

• Symptoms and Adverse Events (Domain 5): Typically captured through clinical interviews and patient reports.

• Participant Disposition (Domain 6): Information on treatment adherence and reasons for discontinuation, gathered via structured interviews or questionnaires.

IMMPACT also highlights the importance of additional domains such as sleep disturbances, fatigue, and the emotional burden of chronic pain, which—although not part of the core domains—are highly relevant for BMS patients. Moreover, emerging evidence suggests a potential link between cognitive impairment and BMS, supporting the integration of cognitive screening into the clinical evaluation. Instruments such as the Mini-Mental State Examination (MMSE) and the Montreal Cognitive Assessment (MoCA) provide valuable screening for cognitive deficits and can guide the need for more comprehensive neuropsychological evaluation when indicated. Incorporating selected IMMPACT-based assessments and cognitive screening into routine BMS management fosters a holistic understanding of the condition, enabling more personalized treatment planning and improving quality of life.

## 11. Conclusions

BMS is a painful condition that necessitates careful evaluation for accurate diagnosis. It is essential to differentiate BMS from other orofacial pain conditions particularly when the pain is unilateral. Additionally, it is crucial to distinguish primary BMS from secondary forms of burning sensations. This distinction is vital as each of these conditions may require different therapeutic approaches. An accurate diagnosis involves considering a range of diagnostic tools including MRI of the brain, blood tests, and cognitive and psychological assessments. These evaluations help to provide a comprehensive understanding of the patient’s condition, leading to appropriate treatment, improved pain management, and enhanced quality of life.
